# Depth information in natural environments derived from optic flow by insect motion detection system: a model analysis

**DOI:** 10.3389/fncom.2014.00083

**Published:** 2014-08-01

**Authors:** Alexander Schwegmann, Jens P. Lindemann, Martin Egelhaaf

**Affiliations:** Department of Neurobiology and Center of Excellence Cognitive Interaction Technology, Bielefeld UniversityBielefeld, Germany

**Keywords:** optic flow, spatial vision, computational modeling, fly, natural environments

## Abstract

Knowing the depth structure of the environment is crucial for moving animals in many behavioral contexts, such as collision avoidance, targeting objects, or spatial navigation. An important source of depth information is motion parallax. This powerful cue is generated on the eyes during translatory self-motion with the retinal images of nearby objects moving faster than those of distant ones. To investigate how the visual motion pathway represents motion-based depth information we analyzed its responses to image sequences recorded in natural cluttered environments with a wide range of depth structures. The analysis was done on the basis of an experimentally validated model of the visual motion pathway of insects, with its core elements being correlation-type elementary motion detectors (EMDs). It is the key result of our analysis that the absolute EMD responses, i.e., the *motion energy profile*, represent the contrast-weighted nearness of environmental structures during translatory self-motion at a roughly constant velocity. In other words, the output of the EMD array highlights contours of nearby objects. This conclusion is largely independent of the scale over which EMDs are spatially pooled and was corroborated by scrutinizing the motion energy profile after eliminating the depth structure from the natural image sequences. Hence, the well-established dependence of correlation-type EMDs on both velocity and textural properties of motion stimuli appears to be advantageous for representing behaviorally relevant information about the environment in a computationally parsimonious way.

## Introduction

Knowing the spatial structure of the surroundings is of crucial importance for many animals, especially if the environment is complex and cluttered. Spatial information is relevant for solving tasks such as collision avoidance, targeting objects, or landmark navigation. Depth information based on far-range mechanisms is of particular importance during fast locomotion in which animals often need to respond to objects when these are still beyond the range of close-up depth-sensing systems, such as stereoscopic vision or tactile sensing (Collett and Harkness, 1982). Motion cues are one powerful source of spatial information, since at least during translatory self-motion the retinal images of environmental objects move faster the closer they are to the observer. Humans experience motion parallax cues, for instance, when looking out of the window of a train and may rely on them when steering a car, especially at high velocities (Vaina et al., 2004).

In particular, flying insects or birds rely on motion cues for spatial vision. These animals were shown to actively shape their visual input by a saccadic flight and gaze strategy that ensures translatory self-motion for most of the flight time: Flight and gaze direction is changed by extremely rapid saccadic turns lasting for less than 20% of flight time; between saccades the gaze is largely kept straight (Schilstra and van Hateren, 1999; van Hateren and Schilstra, 1999; Tammero and Dickinson, 2002; Eckmeier et al., 2008; Mronz and Lehmann, 2008; Boeddeker et al., 2010; Braun et al., 2010, 2012; Geurten et al., 2010; Kern et al., 2012). This peculiar flight and gaze strategy has been interpreted as a means to facilitate extracting spatial information from the image flow on the eyes during translatory intersaccadic motion (Egelhaaf et al., 2012). In accordance with this view, motion sensitive neurons in the visual system of flies as well as of zebra finches were found to represent information about the spatial structure of the environment when stimulated with the retinal image flow that had previously been experienced by free-flying animals. These motion sensitive neurons as well as experimentally validated models of them were found to respond stronger to nearby environmental structures than to more distant ones because the former induced larger retinal velocities (Boeddeker et al., 2005; Kern et al., 2005, 2006; Lindemann et al., 2005; Karmeier et al., 2006; Hennig and Egelhaaf, 2012; Liang et al., 2012; Eckmeier et al., 2013). However, the responses of these motion sensitive neurons are not only affected by retinal velocity, but also reflect the textural properties of moving stimuli, such as their contrast and spatial frequency content (Egelhaaf and Borst, 1993; Straw et al., 2008; Meyer et al., 2011; Hennig and Egelhaaf, 2012). This characteristic feature has often irritated researchers because from the perspective of velocity coding the pattern-dependent response modulations may just reflect a kind of “pattern noise” that deteriorates the quality of the neural representation of pattern velocity (Dror et al., 2001; Rajesh et al., 2005; O'Carroll et al., 2011). Alternatively, however, these pattern-dependent modulations have recently been advocated to be functionally relevant, as they may reflect potentially important information about the surroundings (Egelhaaf et al., 2012). These somewhat contradictory conclusions have been the starting point of the present study. Based on a novel approach, it will integrate both views into a common conceptual framework.

Rather than reconstructing what an animal has seen during behavioral sequences in flight cages and probing motion sensitive neurons or their models with the resulting image sequences, we recorded image sequences in a variety of cluttered natural environments comprising a wide range of spatial, textural, and brightness conditions by moving a high-dynamic range camera on idealized naturalistic trajectories and systematically analyzed by model simulation how spatial information may be represented by the visual motion pathway. How motion information is encoded by fly motion sensitive wide-field neurons has already been analyzed under outdoor conditions. For methodological reasons, these studies could only address the neural responses to rotational displacements of the animal (Egelhaaf et al., 2001; Lewen et al., 2001; Nemenman et al., 2008). Therefore, they had to focus on how reliably self-rotations are represented by the visual motion pathway, but could not address how spatial information is represented. Addressing this issue requires translational self-motion which cannot easily be realized in electrophysiological experiments under outdoor conditions. Therefore, we resorted to model simulations. Moreover, instead of scrutinizing the activity of wide-field neurons, we analyzed the spatio-temporal activity profile of the retinotopic array of elementary motion detectors (EMDs). EMDs subserve the entire visual field by performing local motion measurements before being spatially pooled by the dendrites of wide-field neurons. The model of the motion vision pathway was experimentally validated to a large extent in advance: it can account for the time-dependent output of the fly's visual motion pathway, even under complex behaviorally relevant stimulus conditions (Borst et al., 2003; Lindemann et al., 2005; Shoemaker et al., 2005; Hennig et al., 2011). We will systematically analyze how the activity profile of EMD arrays relates to a variety of features by which natural three-dimensional environments are characterized, such as their local contrast and depth profile.

The key finding of this analysis is that during translatory self-motion, as is characteristic of intersaccadic intervals of insect flight, the motion detection system responds best to the contrast contours of nearby objects in the environment. Hence, both aspects of motion signals, i.e., information about the velocity and texture of environmental structures, are combined in a functionally meaningful way. Although our approach is largely motivated by what we know about visually guided spatial behavior in insects and the underlying mechanism of optic flow computation, the results may generalize to other biological systems as well and may also find an application in technical systems, especially in case of highly efficient and parsimonious computations being an issue.

## Materials and methods

**Figure 1:**
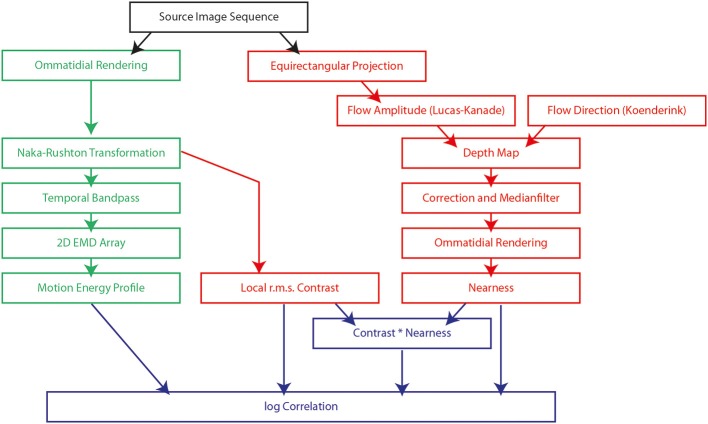
**Flow chart of the model of the fly visual motion pathway (green boxes) and the computations used to obtain information about the environment, such as local contrast, nearness and the contrast-weighted nearness (red boxes).** The model and the computations of environmental information are based on image sequences obtained in natural surroundings (black box). The output of the model of the motion pathway is compared with the determined information about the environment (blue boxes). Each text box indicates a processing element described either by the name of the processing algorithm or the description of the result of the step.

This study is based on a model of the visual motion pathway of the fly and its responses to visual motion sequences as experienced during sequences of translatory self-motion and rapid saccade-like rotations in natural environments with a wide range of depth structures. We analyzed the time-dependent response profiles at different processing stages of the motion pathway and related the output of the array of local motion detectors to a variety of features by which natural three-dimensional sceneries are characterized, such as the local contrast or the nearness to objects. We recorded 37 image sequences in a wide range of different types of natural environments. The latter comprised diverse natural surroundings like cluttered forests, open fields, or shrub land. The recorded image sequences (black box at top of Figure 1) were, on the one hand, used as input to the model of the visual motion pathway (green boxes on the left of Figure 1) and, on the other hand, to extract depth information about the natural environments by computer vision algorithms (red boxes on the right of Figure 1). The depth information determined in this way was used for comparison with the output of the biologically inspired model (blue boxes at bottom of Figure 1).

### Generation of image sequences

For creating the source image sequences (black box at top of Figure 1) we used the same image database as in a parallel study (Schwegmann et al., submitted manuscript). Images were obtained with a high dynamic range (HDR) camera (PhotonFocus MV1-D1312-40-GB-12). The camera was equipped with a panoramic hyperboloidal mirror (Accowle Vision HMN-X15). It had an effective usable resolution with our mirror of 928 × 928 pixels and 12-bit A/D resolution. The resulting image values had a high dynamic range of 1:23,900 sampled in 3,955 intensity steps where the intensity resolution was finer for smaller intensities. To mimic the spectral sensitivity of the flies' photoreceptors R1-R6, that provide the main input of the insect motion vision system (Stavenga, 2002), we limited the camera's spectral sensitivity to wavelengths in the range of 480–550 nm by using a dichroic filter. As a consequence of this filtering and the careful calibration of the CMOS chip of the camera, we could use the linearized digital return values of the camera pixels which are, though being arbitrary units, proportional to light intensity in the green spectral range.

**Figure 2:**
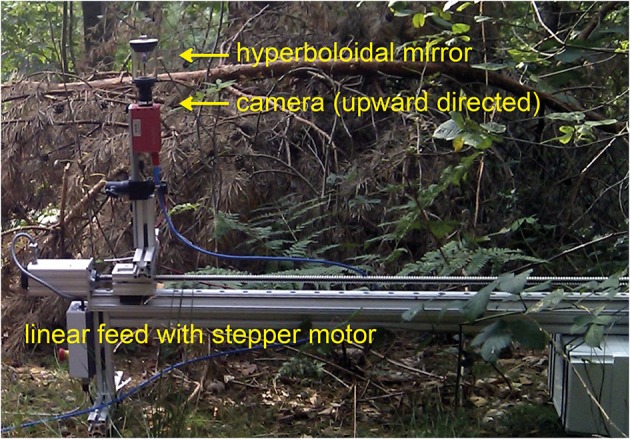
**High-dynamic-range (HDR) camera equipped with a panoramic hyperboloidal mirror and mounted on a motor-driven linear feed.** The camera system could be displaced on a linear track by 1 m. The entire system is shown in one example natural environment.

The camera pointed upwards in the direction of the hyperboloidal mirror (Figure 2). Thus, the final image covered the full 360° azimuth and an elevation ranging from −58° below to 47° above the horizon. In this way, we could capture large parts of the panoramic visual field of an insect with one exposure. Though image resolution drops for patches looking downwards, resolution is still above the resolution of the fly's eye in the entire field of view. To calibrate the mirror geometry for the inverse projection of the image we used a slightly modified version of the omnicam-calibration toolbox by Davide Scaramuzza for MATLAB (MathWorks; version 2010b). The camera was mounted on a custom-made linear feed equipped with a stepper motor and placed 0.5 m above the ground (Figure 2). Camera height was chosen for pragmatic reasons, but was biologically plausible. We recorded images in sequences of 10 mm position steps on a 1-m-linear path. For technical reasons subsequent images were taken at time intervals of 2 s, i.e., at a lower rate than the real motion of an insect at a velocity of, for instance, 1 m/s. Thus, the translational image sequences obtained in this way correspond to those that would have been obtained during real motion only if the visual scenery had not changed (e.g., no brightness changes due to clouds occluding the sun or movements of leaves, etc.). We tried to minimize the resulting artifacts by recording on calm days. Nevertheless, the quality of the calculated nearness maps might have been impaired by these artifacts and have led to a slight underestimation of the correlation between environmental and EMD response parameters (see below). Recording sites were located in different types of natural environments, like cluttered forests, open fields, or shrub land.

To mimic the spatial characteristics of the ommatidial lattice of the blowfly eyes (Petrowitz et al., 2000) we rendered and unwrapped the source images to an equirectangular lattice of square pixels, with each pixel approximating a photoreceptor. The angular distance between these photoreceptors was set to 1.25° and the acceptance angle Δρ of each of them to 1.64°. To mimic the spatial filtering of the insect eye the input was sampled by a two-dimensional Gaussian low-pass filter *F*:

(1) $$F(\phi )\;=\;e^{\left[\frac{-2.77\phi^{2}}{{\left(\Delta \rho \right)}^{2}}\right]}$$

**Figure 3:**
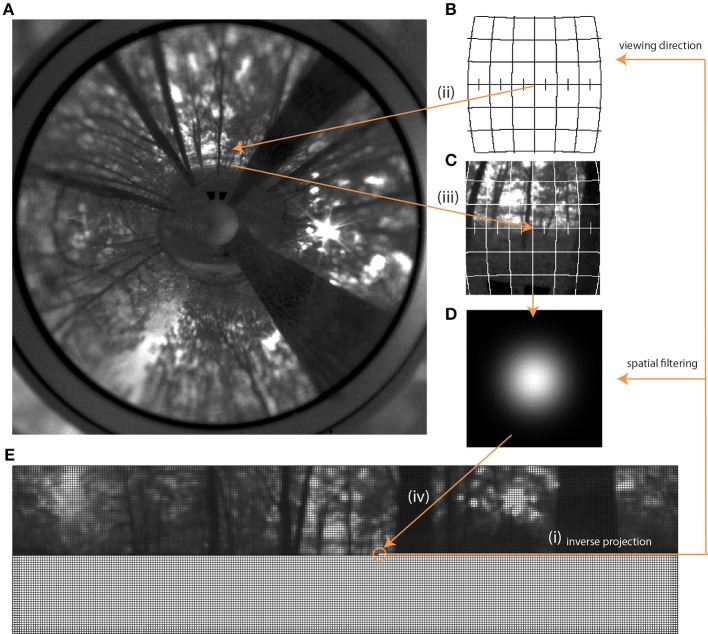
**Rendering process starting with the original panoramic image (A) and leading to the unwrapped image in fly resolution (E).** The first step was to create an empty matrix (bottom part of **E**), each pixel of which was then filled via inverse projection (i). With the given viewing direction for each pixel and the field of view given by the acceptance angle of the photoreceptors, a corresponding sub-projection **(B,C)** in the Lambert Azimuthal Equal Area projection was calculated, also via inverse projection (ii, iii) This sub-projection was then filtered with the Gaussian low-pass filter **(D)** and averaged to obtain the brightness value as input for a given photoreceptor. This value was then written into the corresponding pixel of the matrix (iv) **(E)**.

For each pixel we obtained longitude and latitude in the target projection and computed a piecewise unwrapping of the original ring image into the Lambert azimuthal equal-area projection centered at this longitude and latitude. This type of projection was used because it only leads to a minimal angular distortion for small image patches and is equal-area. To remove aliasing artifacts we used 9 × 9 ordered grid supersampling anti-aliasing before calculating the Lambert patches. We determined the brightness value of the target pixel by computing the sum of the pixel values in the Lambert patch weighted with the values of the Gaussian filter F (Equation 1; Figure 3).

To assess the impact of the environmental depth structure for each image sequence additional versions were rendered in which we eliminated the depth structure virtually. This was done by projecting the original scenery as obtained from the panoramic image taken at the center of the translation track onto the surface of a sphere. Then we virtually moved the camera within this sphere on the same track as was done before in the real environment. We projected by ray-tracing each pixel in each frame on the sphere and calculated the Lambert patch for this region as well as the Gaussian filter correctly rescaled and contorted. The radius of the sphere was set to either 1 or 5 m.

Hence, we had two image sequences for each flight, one with the original depth structure (“*full-depth image sequence”)* and one with a constant depth for all directions (“*depth-equalized image sequence”)*. In the latter case, the images of the sequence are optically distorted the more the camera was displaced from the center of the sphere. Therefore, we only used the responses obtained in the center of the sphere for comparison because the corresponding images were virtually the same in depth-equalized and full-depth version. At this point of the simulated trajectory the only difference between the full-depth and depth-equalized image sequence is the depth distribution.

All image sequences consisted of 100 frames taken at a distance of 10 mm from each other on a straight trajectory. We used the panoramic images because then rotational image displacements can be obtained by software. The simulated movement trajectory started with a 180° yaw turn (1), then a translational phase at 1 m/s (2) using the image sequence as input, then another 180° turn (3) and finally a translation backwards (4) which closes the movement loop. Rotations were rendered according to the dynamics of real blowfly saccades (Schilstra and van Hateren, 1999; van Hateren and Schilstra, 1999), although saccade amplitudes of 180° are beyond the naturalistic range. The same movement trajectory was used for determining the depth-equalized image sequences.

Since we took images only at distances of 10 mm, we did a ten-fold temporal image interpolation from 100 Hz up to 1 kHz to simulate flight speeds of 1 m/s. We employed a *piecewise cubic hermite spline interpolation* for every individual pixel over time. This interpolation seemed to be best suited for this purpose because it is shape-preserving and, therefore, created no overshoots or non-natural sudden or rough intensity changes. After interpolation, all sequences consisted of 2128 frames at a temporal resolution of 1 ms.

### Modeling of visual motion pathway

The model of the visual motion pathway consisted of a sequence of processing steps (green boxes in the flow chart on the left of Figure 1). The motion detector input was non-linearly transformed and temporally filtered to approximate the transfer properties of the peripheral visual system. The non-linear response characteristic of photoreceptors was approximated by Lipetz (1979) or Naka and Rushton (1966) transformation of light intensity *I*:

(2) $$U\;=\;\frac{I^{\alpha }}{I^{\alpha }\;+\;I_{0}^{\alpha }}$$

with *α* being set to 0.7 (Shoemaker et al., 2005), and *I*_0_ being the light intensity corresponding to the mid-response level of the input individually. *I*_0_ corresponds to the average light intensity of each image frame. Since the average brightness values of subsequent images changed only little, *I*_0_ changed only slowly and to a small extent along the motion track in a given environment. *I*_0_ varied much more between different sceneries. The changes in *I*_0_ can be interpreted as a kind of crude global adaptation process to adjust the operating range of the photoreceptors to the respective average ambient brightness conditions. After non-linear transformation the brightness signal was temporally band-pass filtered to mimic the transfer properties of the first optical ganglion. The transfer function was implemented as a serially aligned first-order low-pass filter (τ_*L*_ = 8 ms) and a high-pass filter (τ_*H*_ = 20 ms).

**Figure 4:**
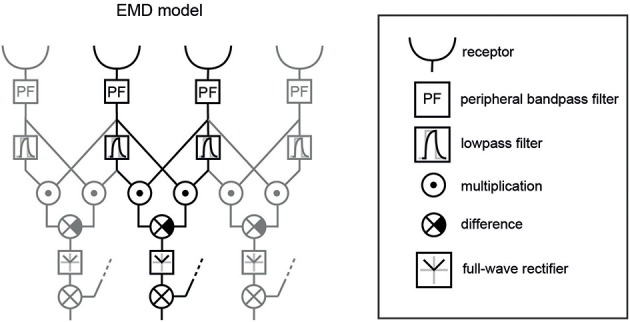
**EMD variant used as core element of the model of the visual motion pathway.** Three neighboring EMDs out of a larger array are shown; one individual EMD is highlighted in black. The receptors include the spatial filter characteristics of the ommatidial lattice of the blowfly eye and the Naka-Rushton transformation of the input signal. Additionally, a peripheral band-pass filter was included to mimic signal transformation performed by lamina monopolar cells. The temporal delay in one branch of each EMD was implemented as a low-pass filter. After having taken the difference between the two mirror-symmetric subunits of an EMD, we applied a full-wave rectifier, since we used the absolute motion signal for correlation with the various image parameters. The summation and the dotted input line at this level indicate the summation of the orthogonally oriented EMDs—horizontal and vertical—resulting in the absolute direction-independent motion energy. Shown elements correspond to the green boxes displayed in Figure 1.

Elementary motion detection was based on two retinotopic arrays of a basic version of correlation-type EMDs (Figure 4). One array consisted of horizontally aligned EMDs, the other of vertically aligned ones. Individual EMDs were implemented by a multiplication of the delayed signal of a receptive input unit with the undelayed signal of a neighboring unit. Only interactions between direct neighbors were taken into account, for both horizontally and vertically aligned EMDs. The delay operator τ_*lp*_ in each half-detector was modeled by a temporal first-order low-pass filter with a time constant of τ_*lp*_ = 40 ms (Shoemaker et al., 2005; Meyer et al., 2011). Each EMD consisted of two mirror-symmetric subunits with opposite preferred directions. Their outputs were subtracted from each other.

For comparison with environmental features (see below) the motion energy was determined for each retinotopic unit by taking the length of the motion vector given by the combination of the responses of a pair of the horizontal hEMD and the vertical vEMD at a given location (*x,y*) of the visual field:

(3) $$absEMD_{\left(x,y\right)}\;=\;\sqrt{vEMD_{\left(x,y\right)}^{2}\;+\;hEMD_{\left(x,y\right)}^{2}}$$

The array of the absolute values of these local motion vectors provided the spatial motion energy profile.

### Estimation of depth structure and local contrast of natural environments

For comparison with the motion energy profile of the arrays of EMDs we determined both local nearness maps and local contrast maps for each image sequence (red boxes in the flow chart on the right of Figure 1).

We computed nearness maps for the analyzed image sequences by using motion parallax cues determined by the Lucas-Kanade algorithm (Lucas and Kanade, 1981). We did the analysis at a resolution of 927 × 251 pixels of equirectangular projections of the original high-resolution images. The images were spatially and temporally smoothed with a Gaussian window using the Lucas-Kanade implementation of the Simulink computer vision system blockset of Matlab. Smoothing was done over 5 subsequent original frames with 0.2 pixels as standard deviation of the spatial filter, 1.5 image frames as standard deviation of the temporal filter and τ = 0.0039 as noise threshold for the calculated eigenvalues (λ).

**Figure 5:**
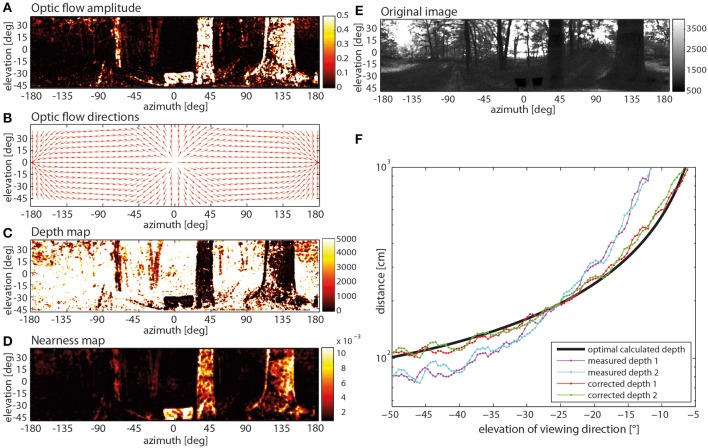
**Steps of determining a nearness map. (A)** Optic flow amplitude determined by way of the Lucas-Kanade algorithm (color code in arbitr. units), **(B)** optic flow directions calculated with the help of a Koenderink algorithm, **(C)** depth map (color code in cm). **(D)** nearness map with corrected depth (color code in cm^−1^), **(E)** original image frame (arbitr. units), **(F)** correction of systematic quadratic error in depth calculation with a comparison of two different measured depth profiles, one measured in the right sideward direction (cyan line) and in the leftwards direction (magenta line), the mathematically correct depth of the environment (black line) and the measured depth after application of the mathematical correction (red and green) were obtained by comparing the measured depths with the correct depth.

When applied to the filtered image sequence, the Lucas-Kanade algorithm provided the optic flow amplitude at each image location (Figure 5A). Since these flow vectors were contaminated with many false directions, they were constrained in their direction by the geometrically correct movement direction: Assuming a stationary scene (Figure 5E) and given the constant displacement between the images, the geometrically correct flow direction could be calculated by the optic flow equation that computes the optical flow vector for a given viewing direction and direction of self-motion (Figure 5B) (Koenderink and van Doorn, 1987). The flow vectors obtained by the Lucas-Kanade method were then projected onto these geometrically correct optic flow vectors. On this basis, the angular displacement due to motion parallax was used to calculate the distance to objects in the environment by triangulation. The resulting depth map for the example is displayed in Figure 5C. Despite all these measures, some noise in the nearness measurements was still obvious (see, for instance, variations of nearness values within the boundaries of the large trees in Figure 5D). Thus, we might have underestimated the correlation values with the motion energy profile to some extent (see below).

Moreover, the depth estimation obtained in this way is affected by a systematic error. This error was determined by applying the described method to a movie generated by computer graphics where every distance to objects in the environment was known (Figure 5F). The error can be minimized by using a calibration obtained by comparing the measured depths (Figure 5F: measured depth 1 and 2) with the known depth structure of the virtual environment (Figure 5F: black line). On this basis, the depth D originally determined can be corrected by

(4) $$D_{c}\;=\;-\;\left(\frac{p_{2}}{2p_{1}}\right)\;+\;\sqrt{{\left(\frac{p_{2}}{2p_{1}}\right)}^{2}+\;\frac{D}{p_{1}}}$$

with correction parameters *p*_1_ = 2.778 · 10^−3^ and *p*_2_ = 0.456 [measured depth 1 and 2 after correction (4): Figure 5F: corrected depth 1 and 2 respectively]. The corrected depth map was smoothed by a 3 pixels x 3 pixels median filter to reduce noise and discard pixels where the distance could not be determined. The smoothed image was down-sampled by applying a 10 × 10 ordered grid anti-aliasing supersampling to the resolution of the photoreceptor lattice. Then, the same Gaussian window (Equation 1), as employed for generating the input image sequence of the motion detection model, was used. This was done to ensure the same visual geometry of the depth map, as used for the EMDs.

To compare the depth structure of the environment with the response profile of the array of EMD pairs we determined the nearness (N) of objects by taking the reciprocal of the corrected depth for each pixel

(5) $$N_{xy}\;=\;\frac{1}{D_{c_{xy}}}$$

The local contrast was calculated as the root mean square (r.m.s.) contrast between each pixel of the image down-sampled to ommatidial resolution, and its eight direct orthogonal and diagonal neighbors. The r.m.s. contrast was calculated by taking the standard deviation of the brightness *I* (x,y) of all pixels *(x,y)* of the local region divided by the mean brightness *I* of the same region (van Der Schaaf and van Hateren, 1996; Brinkworth et al., 2009).

## Results

To investigate what information about the spatial layout of natural environments is represented by the visual motion pathway and, in particular, at its output level we simulated model responses to image sequences obtained on straight tracks in 37 different outdoor environments. We will show a sample image sequence at a given instant of time for the transformations of the visual input along the motion pathway to motivate what aspects of these transformations will be addressed quantitatively for all image sequences.

### Representation of motion in natural sceneries by the visual motion pathway

**Figure 6:**
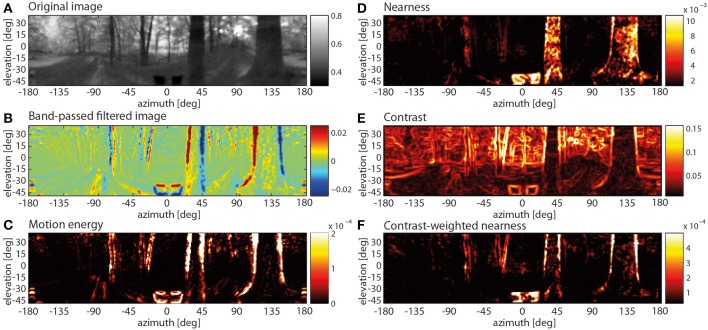
**Spatially resolved responses along the visual motion pathway at one instant of translatory motion through a forest (A–C) and spatial map of three image parameters (D–F). (A)** Original input image (after Naka-Rushton non-linearity; color code in arbitr. units), **(B)** response profile after the temporal band-pass filter in the periphery of the motion pathway (color code arbitr. units), **(C)** motion energy profile obtained from the absolute values of horizontally and vertically aligned EMDs (color code arbitr. units), **(D)** nearness map (color code in cm^−1^), **(E)** local contrast map (color code, local rms contrast), **(F)** contrast-weighted nearness map (color code in arbitr. units).

The example image sequence obtained in a forest environment (Figure 6A) is transformed in a specific way along the different processing stages of the visual motion pathway. This is shown in Figure 6 for the central section of a translation sequence. The time shift of 22 ms caused by the temporal filters of the motion pathway was compensated here and in all subsequent analyses.

It was already at the level of the temporally band-pass filtered activity distribution at the output of the peripheral visual system that mainly the edges of the nearby trees led to positive or negative responses depending on the polarity of brightness change at a given location in the visual field as a consequence of motion. In contrast, the distant parts of the scenery lead to only small responses (Figure 6B). This distance effect was even more obvious in the motion energy profile obtained from the combined output of the arrays of horizontally and vertically aligned EMDs (Figure 6C): Distant background objects, moving slowly on the eyes, create only small or no responses, while near objects are moving at larger velocities and, thus, elicit strong responses. However, these large responses are primarily restricted to the object boundaries.

**Figure 7:**
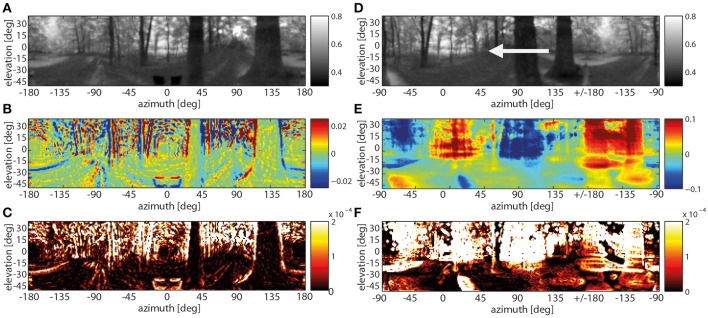
**Spatially resolved responses along the visual motion pathway at one instant of translatory motion after the depth structure of the forest environment being equalized (A–C) and of rotatory motion with a saccade-like velocity profile. (A)** Original input image frame (gray-level code indicating brightness in arbitr. units), **(B)** response activity after the temporal band-pass filter (color code in arbitr. units), **(C)** motion energy profile obtained from the absolute values of horizontally and vertically aligned EMDs (color code in arbitr. units), **(D)** original input image with an arrow indicating the direction of rotation frame (gray-level code indicating brightness in arbitr. units), **(E)** response activity after the temporal band-pass filter—note the different scaling of the color axis in **(B,E)** to cope with the much higher response level during rotations (color code in arbitr., but same units as in **B**), **(F)** motion energy profile for the rotational movement (color code in arbitr., but the same units as in **C**).

In case of the depth structure being equalized, all contrast edges irrespective of their nearness lead to visible responses at both the band-pass filter level and especially at the motion detector output. Without a differentiated depth structure all edges in a scenery move at the same angular velocity across the visual field. Thus the background contours move at a much higher velocity than during translation through the corresponding environment with a pronounced depth structure. In the chosen example, the strongest responses to the depth-equalized image sequence are found in the background as a consequence of high-contrast edges being present there (Figure 7, left panels). This is also true when rotating the entire scenery with a velocity profile resembling a saccadic turn of a fly. Then motion blur emerges as a consequence of the extremely large retinal velocities (Figure 7, right panels).

What stimulus features of the environment are reflected by these characteristic responses, especially at the output of the motion detection system? Since the retinal velocities induced by objects in the environment during translatory motion increase with increasing nearness, a close correlation between the motion energy profile and the nearness map might be expected. However, at first glance both maps differ to some extent. While, for example, in the nearness map (Figure 6D) the entire nearby trees (apart from noise) are leading to large values, the edges of the trees, i.e., regions with a high contrast, mainly lead to strong motion detector responses. Comparing the motion energy profile with the contrast map (Figure 6E) reveals that, although high contrasts are most obvious at object edges, not all regions with high contrasts elicit strong motion responses in the forest environment with its natural depth structure. These results suggest that objects generate strong EMD responses if they (i) are close enough to the observer to elicit large parallax movement and (ii) have a high contrast against their background. Therefore, we determined the contrast-weighted nearness map (Figure 6F). Visual inspection reveals that this map appears to be very similar to the motion energy profile (compare Figures 6C,F).

**Figure 8:**
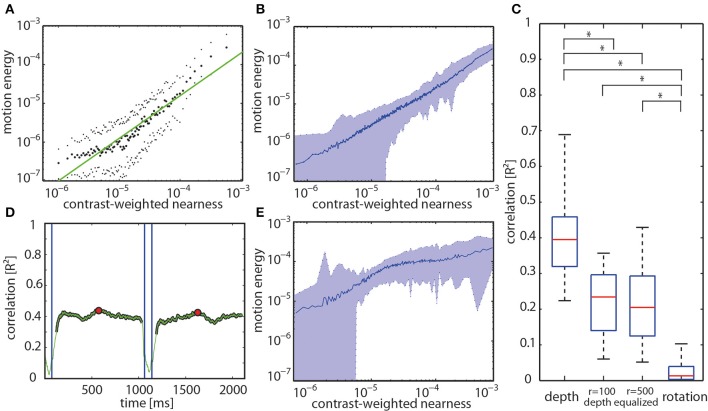
**(A)** Relation between motion energy and the contrast-weighted nearness plotted in a double logarithmic way for the center of the track of the forest scenery shown in Figure 5. **(B)** Relation between motion energy and the contrast-weighted nearness for all full-depth motion sequences of our data base. **(C)** Distribution of correlation values at the center of the tracks is shown by the boxplots for the different stimulus conditions (full-depth movie, depth-equalized movie obtained in two spheres with radius *r* = 100 cm or *r* = 500 cm, and rotation). Boxes indicate the 25th, median (red center line) and 75th percentiles. Whiskers show the minimum and maximum values disregarding the outliers. Asterisks indicate significant differences (*p* << 0.001, paired *T*-test with Bonferroni correction (*n* = 74 per box). **(D)** The time course of the correlation value for entire full-depth movement sequence (green line). Blue vertical lines mark the beginning and the end of a saccade. The red dots mark the center of the translation sequence. **(E)** Relation between motion energy and the contrast-weighted nearness for all depth-equalized motion sequences of our data base.

The hypothesis that the activity profile at the output of arrays of EMDs represents the contrast-weighted nearness or, in other words, the contrast borders of near objects was tested quantitatively for all motion sequences. Plotting the motion energy vs. the contrast-weighted nearness on a double-logarithmic scale reveals a roughly linear relationship between both parameters for the individual forest example mentioned above (Figure 8A) and, on average, for all analyzed natural sceneries (Figure 8B). The slope of this relationship is shallower and, thus, the correlation values are smaller, though still significant, for the depth-equalized motion sequences. This is because there is no depth information, but only contrast left to correlate with (Figure 8E). To quantify these relationships we correlated both parameters at retinal resolution. The correlation between the motion energy profiles and the contrast-weighted nearness maps are much higher for the full-depth than for the depth-equalized image sequences and, especially, for saccade-like rotations (Figure 8C). Moreover, the correlation values are considerably higher than those obtained by correlating the motion energy with either the nearness or the contrast alone (data not shown). The correlation between the motion energy profile and the contrast-weighted nearness reaches a *R*^2^ value of up to 0.7 for some sceneries, whereas the mean value amounts to 0.41 (standard deviation of ± 0.14) reflecting a large scatter between different natural sceneries. This scatter can partly be explained by the large noise in the depth maps, but mainly by the specific properties of the individual sceneries. For instance, image sequences without nearby objects, but a nearby ground consisting of sand with a very low contrast only lead to small EMD responses. In this case, the correlation is small as a consequence of the low signal-to-noise ratio of the nearness estimates.

For translatory motion through the example forest scene with depth structure the *R*^2^ correlation value fluctuates over time around a kind of plateau level. During saccade-like rotations *R*^2^ completely drops toward zero. After the onset of translatory motion, it takes some time for the correlation value to reach its final intersaccadic level after the onset of translatory movement (Figure 8D). Similar time courses were found for the other full-depth motion sequences.

In conclusion, the EMD responses to translatory motion in natural scenes with a clear depth structure depend on the combination of nearness and contrast. This means that the EMDs respond best to the contrast borders of nearby objects. In cases where the nearness is virtually constant, the EMD response essentially depends on local contrast. For being precise, it still depends on nearness because global nearness determines the retinal image velocity for a given translation velocity. As a consequence, smaller global distances result in larger overall responses of the EMDs (data not shown). However, since the nearness is constant for all directions, it does not affect the point-to-point correlation.

### The role of the areas around the foci of expansion and contraction of optic flow fields

The focus of expansion (FOE) and the focus of contraction (FOC) are singularities in the translatory optic flow field where depth cannot be extracted because at these locations there is no retinal image flow. Moreover, since the small retinal velocities close to these singularities might be more affected by noise than the larger velocities in other parts of the visual field, they are likely to reduce the correlation values between motion energy and contrast-weighted nearness. Therefore, we correlated both parameters after excluding the FOE, the FOC, and regions of variable size around these singularities. Moreover, in complementary tests, we kept only those regions around the singular points for correlation.

**Figure 9:**
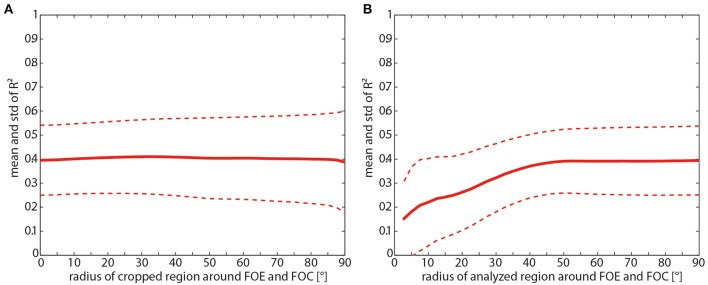
**(A)** Consequences of removing the foci of expansion (FOE) and contraction (FOC) for the mean correlation (solid line) and its standard deviation (dashed line) between the motion energy profile and the contrast-weighted nearness map as a function of the radius of the cropped region centered at the FOE and FOC. **(B)** Consequences of keeping only the FOE and FOC for the mean correlation (solid line) and its standard deviation (dashed line) as function of the radius of the region centered at the FOE and FOC.

Removing the FOE and the FOC as well as the surrounding areas does not increase the correlation at all (Figure 9A). On the other hand, with only taking the singularities and the circumjacent area into account and decreasing the size of this area, the correlation values started to decrease at a radius around the foci smaller than 40° (Figure 9B).

### The role of spatial pooling

**Figure 10:**
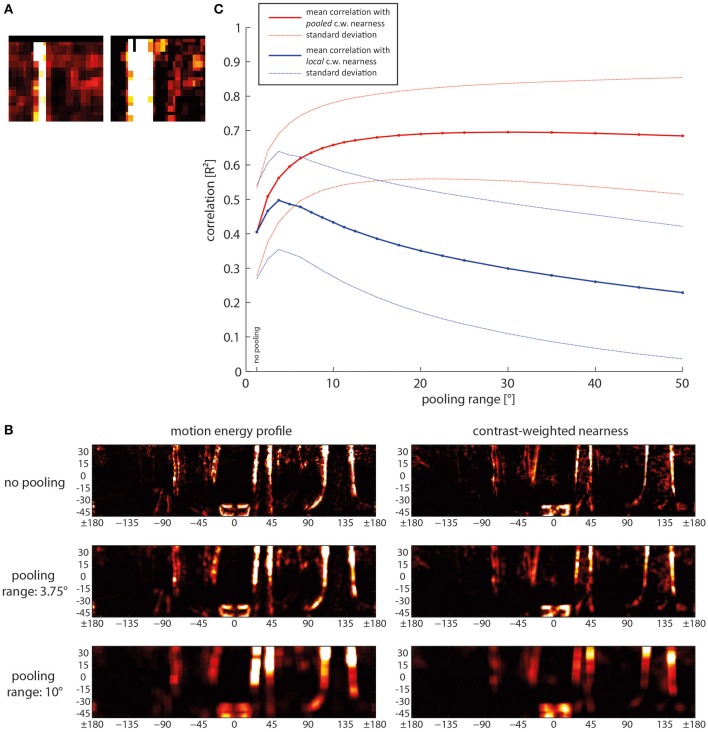
**Consequences of spatial pooling. (A)** Close-up of a small area of the motion energy profile (left) and the corresponding contrast-weighted nearness map (right) to illustrate differences in detail. **(B)** Mean correlation (solid lines) and standard deviations (dashed lines) as a function of pooling range. Red lines: Correlation of the pooled motion energy profile with the pooled nearness map. Blue line: Correlation of the pooled motion energy profile with local non-pooled nearness map, indicating the reduction of localizability with increasing pooling range. **(C)** Examples of motion energy map (left) and contrast-weighted nearness map (right) for different pooling ranges, ranging from no pooling in the upper row over small-range pooling of 3.75° in the middle row to a pooling range of 10° in the bottom row. c.w., contrast weighted.

What are the consequences of spatial pooling on the correlation of motion energy and contrast-weighted nearness? We expected some increase with spatial pooling because we observed, for instance at the edges of trees, small shifts between pixels with large contrast-weighted nearness values and the corresponding pixels of high activity in the motion energy profile (Figure 10A). Therefore, spatial pooling might be an easy way to raise the correlation between motion energy and contrast-weighted nearness and, thus, the reliability of local spatial information. However, this might be possible only at the expense of resolution and localizability of the available information. The effect of pooling was analyzed by convolving the data matrix with a square, uniformly-weighted filter of a size given by the pooling range before computing the correlation (for examples, see Figure 10B).

Spatial pooling over a small range already increases the reliability of the local contrast-weighted nearness information to a great extent (Figure 10C, bold red line). However, spatial pooling over a range larger than approximately 10° does not further increase the correlation between motion energy and contrast-weighted nearness. For this analysis we computed pooling for both the motion energy profile and the contrast-weighted nearness map.

To increase reliability spatial pooling over the direct and second neighbors is already effective. A further increase of the pooling size raises the correlation of the motion energy with contrast-weighted nearness only very slightly. Instead, localizability of the information decreases (Figure 10B, bold blue line). This was determined by keeping the contrast-weighted nearness information at high resolution (Figure 10C, top right image), while the motion energy profile was pooled in the same way as before. Then, the reduction of localizability is reflected in a decrease in correlation. We found an optimal pooling size of 3.75°, which corresponds to the receptive unit and its nearest neighbors. For larger pooling ranges the correlation indicating localizability decreases.

## Discussion

To navigate through cluttered environments an animal has to extract information about the spatial structure of its immediate surroundings. In fast-flying insects, motion parallax, i.e., the relative motion of objects on the eyes induced during translatory self-motion, can be assumed to be the main source of depth information. Motion parallax cues depend on the distance of environmental objects to the eyes and, thus, provide depth information. Veridical depth information can only be obtained in this way if the local retinal velocities in the respective regions of the visual field can be extracted from the retinal motion patterns. This, however, is by far no trivial task both for biological and technical systems.

Much is known about the mechanisms of motion detection in insects and especially in flies at the level of neural circuits due to great methodological advances during recent years, (e.g., Borst, 2009; Reiff et al., 2010; Maisak et al., 2013; Silies et al., 2013; Takemura et al., 2013; Hopp et al., 2014; Mauss et al., 2014; Meier et al., 2014; Strother et al., 2014). The overall performance of these circuits can be lumped together and has been explained for long by a computational model of local motion detection, the correlation-type elementary motion detector (EMD), which also formed the basis of our study (Reichardt, 1961; Borst and Egelhaaf, 1989, 1993; Egelhaaf and Borst, 1993). Based on this model, the time course of the responses of fly motion sensitive neurons can be well described, even for the dynamically complex stimulus conditions that are encountered during free-flight sequences (Lindemann et al., 2005; Hennig et al., 2011; Hennig and Egelhaaf, 2012).

Despite the detailed knowledge at the cellular and computational level, the functional significance of the information provided by these movement detectors has not been clear yet. This statement may sound surprising, given the conventional wisdom that motion detectors should represent velocity information as veridically as possible. However, it is known for long that the performance of the insect motion detection system systematically deviates from this expectation. Although EMDs exploit the different speeds of objects and, thus, may also obtain information about the depth structure of the environment, they are also sensitive to textural features of the environment (review: (Egelhaaf and Borst, 1993): Their responses increase—within a certain range—with contrast and are most sensitive to spatial frequencies in an intermediate range, but do not respond much to the low frequencies that are most prevalent in natural sceneries (Meyer et al., submitted manuscript).

Although this pattern dependence of EMD responses has often been concluded to be a kind of “pattern noise” of a somehow deficient biological motion detection mechanism (Dror et al., 2001; Rajesh, 2005; O'Carroll et al., 2011), we conclude that this pattern dependence may make sense from a functional perspective at least during translatory self-motion in cluttered natural environments. Several previous studies already probed the insect motion vision system with natural sceneries. This was done either in electrophysiological experiments under outdoor conditions, while the entire preparation was rotated around its yaw axis (Egelhaaf et al., 2001; Lewen et al., 2001; Nemenman et al., 2008) or in electrophysiological studies under lab conditions and by model analyses while presenting moving natural images (Straw et al., 2008; Wiederman et al., 2008; Brinkworth et al., 2009; Barnett et al., 2010; Meyer et al., 2011; O'Carroll et al., 2011; see however, Boeddeker et al., 2005). However, almost all of these studies only employed motion sequences that did not contain any depth information and, thus, differed tremendously from what an animal experiences when flying around in natural environments. In contrast, we systematically employed stimulus sequences that contained the natural depth information of a large number of cluttered environments and compared the resulting activity profiles of arrays of EMDs with those obtained with stimulus sequences where the depth structure of the environment was removed. In this way we could show that EMD arrays do not respond best to the retinal velocity and, thus, to the nearness of environmental structures *per se*, but to the contrast-weighted nearness, or in other words, to the nearness of high-contrast contours of objects. This conclusion holds true as long as the translational velocity varies only little and, thus, does not induce time-dependent response changes just as a consequence of the changes of self-motion. This condition is met to a large extent during individual intersaccadic intervals of insects (Schilstra and van Hateren, 1999; van Hateren and Schilstra, 1999; Kern et al., 2012). Our model deviates from the response properties of the insect motion detection pathway for high contrast values. In this case, the responses of the neuronal counterparts of our model EMDs saturate and, thereby, depend less strongly on contrast. This effect may reduce the correlation of the motion responses with contrast-weighted nearness and increase the correlation with nearness alone. However, we expect this to be only a quantitative effect. From a qualitative point of view, the motion energy computed by insect motion detection will represent contrast as well as nearness, and thus the contours of nearby objects, even in a high-contrast regime.

Another feature of EMDs may interfere with their ability to convey spatial information. The responses of EMDs increase with velocity only in a limited velocity range, beyond which they decrease again (Egelhaaf and Borst, 1993). Hence, relatively unambiguous nearness information can only be provided as long as the response amplitude depends monotonically on retinal velocity. At least flying insects, such as bees, flies and moths, appear to deal with this characteristic of EMDs by a behavioral strategy: By adjusting their flight speed, these animals keep the optic flow on their eyes in a range in which the responses increase monotonically with increasing velocity and decrease with decreasing velocity. Accordingly, the animals decelerate when the translational optic flow increases, for instance, while passing a narrow gap or flying in a narrow tunnel (Srinivasan and Zhang, 2004; Srinivasan, 2011; Egelhaaf et al., 2012). This strategy, however, implies that a given range of optic flow amplitudes corresponds to different nearness ranges, depending on flight speed. In other words, the spatial range that can be encoded in the monotonic range of the motion detection system scales with locomotion velocity. Under spatially constrained conditions in which flies were observed to fly at translational velocities of only slightly more than 0.5 m per second, the spatial range within which significant distance dependent intersaccadic responses are evoked amounts to approximately two meters (Kern et al., 2012; Liang et al., 2012). From an ecological point of view, this scaling of the spatial range with flight speed is economical and efficient: A fast moving animal should initiate, for instance, a collision avoidance maneuver earlier and at a greater distance from an obstacle than when moving slowly. Collision avoidance thus may be triggered at a similar time to collision for different translation velocities.

When interpreting optic flow amplitudes during translatory motion with respect to nearness information, the characteristic geometry of optic flow needs to be taken into account. Even when moving in the center of a sphere where the distances in all directions are the same, the optic flow varies systematically across the visual field: it increases from the direction of heading, where it is zero, toward the lateral visual field, and then decreases again (Koenderink, 1986). This implies that equally distant and equally contrasted objects lead to the strongest responses when they are directly at the side. Hence, nearness information that can be gathered from optic flow needs to be scaled according to the retinal location relative to the direction of motion. Such a scaling might be accomplished by appropriately weighing the spatial sensitivity of the motion detectors in the different eye regions. However, such differential weighing is not required, if the optic flow just within a given limited area of the visual field is used for solving a particular behavioral task. Collision avoidance might be such a task: Here, blowflies have been concluded to employ only the optic flow in the fronto-ventral visual field to determine the direction of an evasive turning response (Kern et al., 2012).

After a change from a saccadic rotation to an intersaccadic translational movement, it takes some time for the movement detector response to reach a kind of steady-state level (see beginning of trace in Figure 8D). This finding indicates that the first centimeters of a translational sequence cannot be used by the animal for a reliable estimation of environmental parameters. Therefore, a minimal duration of translatory flight segments of about 50–70 ms—depending on the time constants of the motion vision system—is required for the animal to be able to achieve reliable nearness information from the optic flow. Indeed, intersaccades of flies tend to have durations in this range, even in small flight arenas where saccadic direction changes need to frequently be generated to avoid collisions with the arena wall (Schilstra and van Hateren, 1999; van Hateren and Schilstra, 1999; Kern et al., 2012). Hence, it appears that even under such constraints the duration of intersaccadic intervals is long enough to allow for extracting spatial information from the optic flow patterns on the eyes.

Spatial pooling of responses of neighboring EMDs could be shown to considerably increase the reliability with which the boundaries of nearby objects are represented in the motion energy profile. Pooling of the direct and second neighbors is already sufficient. Increasing the pooling area further does not increase the contrast-weighted nearness information significantly, but reduces the localizability of environmental features.

Most knowledge of the representation of motion information in insects is based either on computational modeling or on recordings from wide-field neurons that spatially pool the outputs of local motion sensitive elements across extended parts of the visual field (Borst and Haag, 2002; Egelhaaf, 2006; Taylor and Krapp, 2008; Borst, 2009; Borst et al., 2010; Egelhaaf et al., 2012). Such wide-field cells are not only known in insects, but also in other animals such as birds and mammals (e.g., Simpson, 1984; Frost et al., 1994; Duffy, 1998). Although these cells have large, but still spatially restricted receptive fields, it is suggested that to some extent they represent—apart from genuine motion information—information about the environment. The spatial range over which this information is pooled is likely to depend on the behavioral task the respective neurons are involved in. One obvious task of motion vision systems is to provide the animal with self-motion information, i.e., information about the rotational and translational components of its own movements. Self-motion information is particularly relevant for animals moving in three-dimensional space, such as flying insects and birds, and is contained in the behaviorally generated optic flow fields. Deviations from an intended direction and/or velocity of self-motion are thought to be detected by the motion vision pathway and compensated by optomotor responses. The underlying mechanisms that extract the relevant information from the optic flow patterns on the eyes should ideally be independent from the textural and spatial layout of the environment and only reflect the self-motion. Spatial integration over large parts of the visual field enhances the specificity of the system for different types of self-motion (Hausen, 1981, 1982; Krapp et al., 1998, 2001; Dahmen et al., 2000; Franz and Krapp, 2000; Horstmann et al., 2000; Karmeier et al., 2003; Franz et al., 2004) and cancels out, at least to some extent, environmental information, such as time-dependent modulations in the local motion responses resulting from differences in nearness and/or texture (Meyer et al., 2011; O'Carroll et al., 2011). Although many motion sensitive neurons that are thought to provide information about rotational self-motion have large receptive fields, their receptive fields are spatially clearly restricted. As a consequence, these cells are far from being ideal detectors of self-rotation, as they show clear pattern-dependent response modulations (Meyer et al., 2011; O'Carroll et al., 2011; Schwegmann et al., submitted manuscript). On the other hand, the large, though spatially restricted, receptive fields of these wide-field motion sensitive cells make them by no means ideal sensors for information about the surroundings and, in particular, its spatial layout—at least not on a fine spatial scale. At best, these cells are able to represent the average nearness and/or the average pattern properties in relatively large parts of the visual field. The time course of their output signals reflects environmental pattern and spatial properties during translatory intersaccadic flight phases (Kern et al., 2005; Karmeier, 2006; Hennig and Egelhaaf, 2012; Liang et al., 2012). It is still a controversial issue whether and for what computational purpose this information is employed in visually guided orientation behavior, such as in collision avoidance (Tammero and Dickinson, 2002; Lindemann et al., 2008; Kern et al., 2012; Lindemann and Egelhaaf, 2013).

These considerations lead us to suggest that the size of the receptive fields of insect wide-field neurons—but potentially also of motion sensitive neurons in other systems—represents a kind of compromise between various demands, which makes them suitable to play a role in a variety of computational tasks, such as self-motion estimation, spatial navigation or collision avoidance, although they may not be optimally tuned to any of these tasks on their own. Combining the outputs of such “suboptimal” neurons in different task-dependent constellations might be a more parsimonious strategy in terms of expenditure of neural hardware than having a larger sample of cells that are specifically tuned to each individual task.

In conclusion, we have shown that during translatory locomotion the largest responses of the motion detection system are induced by contrast borders of nearby objects. Hence, from a functional perspective this conclusion pertains much to the characteristic flight and gaze strategy of insects (see above). Here, the animals essentially move straight for more than 80% of flight time and change their direction by interspersed saccadic turns of variable amplitude. Since translation velocity does not change much during intersaccadic intervals (Schilstra and van Hateren, 1999; van Hateren and Schilstra, 1999; Boeddeker et al., 2010; Kern et al., 2012), modulations in the output of the motion detection system reflects discontinuities in the depth structure and/or textural properties of the environment or, in other words, the contrast borders of nearby objects, rather than changes in the velocity of self-motion. Thus, what has been conceived by common wisdom to be a deficiency of the insect motion detection system may turn out to be a means that allows—in combination with the active flight and gaze strategy—to parse the environment into near and far and, at the same time, enhance the representation of object borders in a computationally extremely parsimonious way.

### Conflict of interest statement

The authors declare that the research was conducted in the absence of any commercial or financial relationships that could be construed as a potential conflict of interest.
